# Faecal Short Chain Fatty Acids Profile is Changed in Polish Depressive Women

**DOI:** 10.3390/nu10121939

**Published:** 2018-12-07

**Authors:** Karolina Skonieczna-Żydecka, Elżbieta Grochans, Dominika Maciejewska, Małgorzata Szkup, Daria Schneider-Matyka, Anna Jurczak, Igor Łoniewski, Mariusz Kaczmarczyk, Wojciech Marlicz, Maja Czerwińska-Rogowska, Justyna Pełka-Wysiecka, Karolina Dec, Ewa Stachowska

**Affiliations:** 1 Department of Biochemistry and Human Nutrition, Pomeranian Medical University, 71-460 Szczecin, Poland; karzyd@pum.edu.pl (K.S.-Ż.); dmaciejewska.pum@gmail.com (D.M.); sanprobi@sanprobi.pl (I.Ł.); mczerw@pum.edu.pl (M.C.-R.); karolinatdec@gmail.com (K.D.); ewast@pum.edu.pl (E.S.); 2 Department of Nursing, Pomeranian Medical University, 71-210 Szczecin, Poland; szkup.m1@gmail.com (M.S.); daria.schneidermatyka@gmail.com (D.S.-M.); 3 Department of Clinical Nursing, Pomeranian Medical University, 71-210 Szczecin, Poland; jurczaka@op.pl; 4 Department of Clinical and Molecular Biochemistry, Pomeranian Medical University, 70-111 Szczecin, Poland; mariush@pum.edu.pl; 5 Department of Gastroenterology, Pomeranian Medical University, 71-252 Szczecin, Poland; marlicz@hotmail.com; 6 Department of Psychiatry, Pomeranian Medical University, 71-460 Szczecin, Poland; wysiecki@wp.pl

**Keywords:** short chain fatty acids, fiber, depression, pharmacotherapy, microbiota, gut-brain axis

## Abstract

Short chain fatty acids (SCFAs) being produced during fermentation of non-digestible polysaccharides are regulatory compounds with the potential to influence inflammatory, as well as emotional state and cognition through the gut–brain axis. We analyzed the association between stool concentration of SCFAs (acetic acid (C 2:0), propionic acid (C 3:0), isobutyric acid (C 4:0 *i*), butyric acid (C 4:0 *n*), isovaleric acid (C 5:0 *i*) valeric acid (C 5:0 *n*), isocaproic acid (C 6:0 *i*), caproic acid, and (C 6:0 *n*) heptanoic acid (C 7:0)) and depressive symptoms among women and looked for the potential confounders of microbiota byproduct synthesis. We enrolled 116 women aged 52.0 ± 4.7 years and recognized depression in 47 (40.52%). To analyze the emotional state, Beck’s Depression Inventory (BDI) was used. We assessed SCFAs content by means of gas chromatography. Fiber intake was estimated using parts of food frequency questionnaire. The content of acetic acid was significantly lowered compared to non-depressed women (median {IQR}: 29.49 {20.81} vs. 34.99 {19.55}, *p* = 0.04). A tendency toward decreased level of propionic acid was noticed (median {IQR}: 16.88 {9.73} vs. 21.64 {12.17}, *p* = 0.07), while the concentration of isocaproic acid was significantly increased in (median {IQR}: 0.89 {1.15} vs. 0.56 {0.95}, *p* < 0.01) comparison to matched healthy subjects. We found negative correlations between acetate, propionate, and Beck’s score (r = −0.2, *p* = 0.03; r = −0.21, *p* = 0.02, respectively). Statistically significant correlations between acetate and propionate and BDI somatic score (r = −0.21, *p* = 0.01; r = −0.17, *p* = 0.03), as well as correlations regarding isocaproic and both cognitive/affective (r = 0.37, *p* = 0.0001) and somatic (r = 9.37, *p* < 0.001) scores were found. Women who declared current usage of lipid-lowering and thyroid drugs in the past, had higher content of C6:0-i (Users; median {IQR}: 1.91 {3.62} vs. non-users; 0.55 {0.67}; *p* = 0.0048).and lower of C2:0 (Users; median {IQR}: 23.07 {12.80} vs. non users 33.73 {21.44}; *p* = 0.041), respectively. No correlations regarding SCFAs concentration and fiber intake were found. We concluded that SCFAs may potentially contribute to depression phenotype, however, due to the small size of groups suffering from moderately heavy (*n* = 5) and severe (*n* = 7) depression, the conclusion should be treated with caution. Pharmacotherapy of hyperlipidemia and thyroid disease might affect SCFAs synthesis. Studies with more participants are required.

## 1. Introduction

Depression is one of the most common mental disorders, diagnosed mainly in women. According to recent data from the World Health Organization (WHO), depression affects up to 300 million people worldwide, especially between the age of 20–40 years [1]. It was estimated that depression was diagnosed in nearly 10% of polish population [2]. The disease seriously impairs the quality of life of patients and is one of the leading causes of social disability. Untreated depression is associated with an increased risk of morbidity and mortality, including suicide. WHO reports that every year, 800,000 people die as a result of self-assassinations. [1].

Multiple theories of depression exist, among them monoamine deficiency [3] and neurogenesis disruption are two predominant ones [4]. Recently it was proved that the alterations within the gut microbiota composition—referred to as dysbiosis and consequently skewed production of microbiota byproducts—may play a role in the genesis of inflammatory state in situ but also affect in the bidirectional communication between gut and brain [5,6,7]. To close the vicious circle, inflammation has been recently implicated in the origin of depression [8]. Moreover, as environmental factors, predominantly diet, affect gastrointestinal microbiota [9,10], the evaluation of its composition may serve as a novel diagnostic tool to assess susceptibility to certain diseases [11].

Microbiota alterations between depressive and non-depressive subjects have been confirmed, however results are conflicting [12]. A few studies conducted in rodents proved that microbiota composition is altered during depression. First, gut microbiota richness and diversity were found to be depleted [13]. Second, transplantation of stool sampled from people with major depressive disorder (MDD) to germ free rodents led to behavioral and physiological traits of depression [14]. Kelly et al. [13] analyzed the gut microbiota composition in 34 patients with depression and found that abundance of *Prevotellaceae* was lowered in contrast to the relative proportion of *Thermoanaerobacteriaceae* found to be elevated compared to healthy controls. Also, *Eggerthella, Holdemania, Gelria, Turicibacter, Paraprevotella*, and *Anaerofilum* genera counts were overrepresented, whereas *Prevotella* and *Dialister* decreased. One year earlier, Jiang et al. [15] demonstrated the elevation of *Enterobacteriaceae* and *Alistipes*, in parallel with the decrease in *Faecalibacterium* spp. in depressive persons. Consequently, the dysbiotic changes were accompanied by altered amino acid and carbohydrate metabolism [14,16].

Nonfermentable polysaccharides, e.g., fiber, passes to the cecum and large bowel to be anaerobically fermented by gut microbiota into multiple metabolites, with SCFAs being the most representative ones. Therefore, the analysis of the SCFAs concentration could serve as an indirect way to analyze microbiota composition. Although SCFAs include a group of compounds built out of 1-6 carbon, C2–C4 (namely acetate, propionate, and butyrate respectively) account for over 95% of the total SCFAs pool [17]. It was elegantly shown that SCFAs affect host physiology [18]. The molecules are required within the intestinal anaerobic niche to balance the production of redox equivalent [19], to maintain gut barrier integrity [20], to synthesize gut hormones [21], to regulate epigenetic processes [22], and other pathways of human host physiology [18], including gut–brain signaling [23]. Of note, Naseribafrouei et al. [24] demonstrated that the *Oscillibacter* genus was associated with the depressive phenotype and importantly its main SCFA, valeric acid, resembled GABA, thus it could potentially be involved in the pathogenesis of depression. Indeed, as elegantly reviewed by Oleskin and Shenderov [25], SCFAs acting within intestinal endocrine cells stimulate the production of histamine, serotonin, 5-aminovaleric acid, and γ-aminobutyric acid, all being neuroactive molecules.

As gut microbiota alterations in the course of depression were already established, we hypothesized that microbial products of metabolism may also be skewed in patients diagnosed with depression. Therefore, in the present study we aimed to (1) analyze stool concentration of SCFAs in women with depression; (2) look for the link between SCFAs and emotional state in Polish healthy women; and (3) find potential confounders of SCFAs production in the study group.

## 2. Materials and Methods

### 2.1. Data Collection

A local paper advertisement and publicly placed posters were used to recruit women for the study. Participants were nonrandomly selected by the chain-referral sampling method. The inclusion criteria included: females, age between 45–60 years, the lack of diagnosed cancer and inflammatory diseases, non-use of antibiotics, glucocorticosteroids, and pro/prebiotics within one month preceding the beginning of a study. Deliberate written consent to take part in the survey was necessary. Anyone who failed to meet these criteria was not qualified to participate in the project. Finally, the study sample consisted of 116 women, all being inhabitants of the West-Pomeranian Province (Poland). All subjects gave their informed consent to participate in the study. The study was conducted in accordance with the Declaration of Helsinki, and the protocol was approved by the Bioethical Commission of the Pomeranian Medical University in Szczecin, Poland (resolution no. KB-0012/181/13 and KB-0012/104/11).

### 2.2. Surveys

The data collection procedure covered an interview with a study participant to collect basic information concerning sociodemographic and health-related data. Three nurses were interviewing women who volunteered to participate in the study. The staff were trained to conduct the interview in identical conditions (e.g., identical wording, order of questions) and supervised by a main investigator (E.G.). The participants provided information on their education, place of residence, marital status, professional activity, as well as the use of stimulants and medications especially due to hypertension, diabetes, and coronary artery disease. The history of taking drugs was also recorded. No anthropometric data was collected.

The self-esteem Beck depression inventory (BDI-I) [26] was completed by the study participants during the meeting with the researcher. The questionnaire was shown to possess optimal psychometric properties among them internal-consistency estimates (a) equal to 0.86 for psychiatric patients and 0.81 for healthy individuals [27]. BDI-I scale is a 21-item self-report questionnaire with multiple-choice questions to evaluate the presence of depressive symptoms and to track their intensity. A four-point Likert scale (0–3; on the basis of the symptom severity) is used to rate each of BDI-I item with a time frame covering past two weeks. The higher score, the more severe depressive symptoms. The total BDI-I score up to 11 indicates no depressive symptoms, 12–19 mild depression, 20–25 moderate depression, and 26–63 heavy depression [27].

Food frequency questionnaire (FFQ) was used to obtain frequency and portion size information on fiber consumption. Thus, in the present study, data on the following products consumption were evaluated: whole-meal bread, refined bread, coarse groats, fine cereal, ready-to-eat breakfast products, stone fruit, kiwi, tropical fruits, berries, bananas, apples, avocados, olives, dried fruit, processed fruit, cruciferous vegetables, yellow-orange vegetables, green vegetables, tomato, cucumber, root vegetables, fresh and dry legume seeds, nuts, grains, fruit juices, and vegetable juices. Women were asked to mark the consumption over past year. For each product, the average fiber content per 100 g of the product was determined. By means of the Diet5d software(version 5.8.2, Warsaw, Poland), the fiber content per 100 g in individual products was determined, e.g., coarse grains- the fiber content in buckwheat groats was selected: buckwheat, pearl barley, brown rice, etc.; coarse grains per 100 g of product. Next, the fiber content per 100 g was multiplied by the time the portion was consumed (usually it was 1 serving, e.g., 1 cup, 1 fruit, 1 slice, and in the case of groats or cruciferous vegetables, the average portion consumed by women). In this way, the fiber content was obtained in the product portion. The content of fiber in the portion was multiplied by the frequency of each food consumption. To obtain such data, ranks marked within the FFQ were replaced according to the adopted rule: never–0 times per day; 1/month–0.016 times per day; a few/month–0.08 times per day; several /week–0.43 times per day; every day–1 times per day; several times a day–2.5 times per day. Conversions were assessed as described earlier [28].

### 2.3. Sampling

We collected ulnar venous blood using the Vacutainer system to evaluate basic biochemical parameters, such as the serum levels of insulin, glucose, total cholesterol, low-density lipoprotein cholesterol (LDL), high-density lipoprotein cholesterol (HDL), triglycerides (TAG), and C-reactive protein (CRP). The blood was collected by qualified nurses and delivered to the laboratory in accordance with the relevant rules and procedures.

Every woman entering the survey was asked to collect a stool sample into a screw-capped collection container using a plastic holder to use the collection container in the toilet. Study participants were advised not to use laxatives, including mineral oil and castor oil, eat synthetic fat substitutes, or take fat-blocking nutritional supplements. If a barium enema was introduced, women were asked to wait 48 h before collecting the feces. Women were sampling feces after overnight fasting, to establish a common reference point for food intake, a factor determining SCFAs synthesis. After the stool was collected, women were delivering the samples within 24 h to our laboratory. The samples were then stored at −80 °C until the analyses.

### 2.4. SCFAs

#### 2.4.1. Isolation of SCFAs

A 0.5 g fecal sample was suspended in a tube containing 5 mL of water and mixed intensively for 5 min. Using 5 M HCl solution, the pH of suspension was adjusted to 2–3. The samples were then shaken for 10 min and centrifuged for 20 min at 5.000 rpm. Subsequently, the supernatant was filtered (Ø 400 µm) and transferred to a chromatographic vial for gas chromatography analyses.

#### 2.4.2. Gas Chromatography

The following SCFAs were analyzed: acetic acid (C 2:0), propionic acid (C 3:0), isobutyric acid (C 4:0 *i*), butyric acid (C 4:0 *n*), isovaleric acid (C 5:0 *i*) valeric acid (C 5:0 *n*), isocaproic acid (C 6:0 *i*), caproic acid (C 6:0 *n*), and heptanoic acid (C 7:0). However, in our study we evaluated C2–C6:0 acids, as these were reported to be produced by gut microbiota. Chromatographic analyses were carried out using the Agilent Technologies 1260 A GC system with a flame ionization detector (FID). Fused-silica capillary column with a free fatty acid phase (DB-FFAP, 30 m×0.53 mm×0.5 um) was used. The carrier gas was hydrogen at the flow rate equal to 14.4 mL/min. The initial temperature (100 °C) was maintained for 0.5 min, then raised to 180 °C with ramping of 8 °C/min to be constant for 1 min. Subsequently, the temperature was increased to 200 °C (ramping 20 °C/min), to finally reach 200 °C and be sustained for 5 min. The injection volume was 1 μL and the run time of a single analysis was 17.5 min.

### 2.5. Statistical Analysis

Shapiro–Wilk normality test was done to assess the distribution of continuous variables. Nonparametric Mann–Whitney test was used to compare the concentration of SCFAs between depressed women and control subjects. We correlated the total score of BDI-I and SCFAs level by means of Spearman rank correlation coefficient analyses. Multiple linear regression model was used to assess the relationship between SCFAs, depression, and pharmacotherapy. All SCFAs were log-transformed prior to the regression analysis. The acceptable probability of error for the first type was assumed to be equal to 0.05. These statistical analyses were performed using the StatView computer software version 5.0 (SAS Institute Inc., Cary, NC, USA).

## 3. Results

### 3.1. The Prevalence of Depression

There were 116 women aged 52.0 ± 4.7 years old enrolled. The mean score in Beck’s scale was 8.7 ± 9.4 points (Min: 0; Max: 38; IQR: 16) which allowed us to recognize mild depression in 35 (30.17%), moderately heavy in 5 (4.31%), and severe in 7 (6/3%) women. Due to relatively small number of participants with depressive symptoms, they were combined into one group. Then, the depressed group consisted of 47 (40.52%) subjects. Sociodemographic and health-related data with respect to depressive symptoms are summarized in Table 1. No statistically significant differences between depressed and non-depressed individuals were found.

### 3.2. SCFAs Concentration Correlates to the Severity of Depressive Symptoms

The mean concentration of SCFAs in a whole study group was 104.38 ± 41.47 µmol/g of faeces (Min: 40.85; Max: 257.28; median: 99.03; IQR: 59.01). We found that non-depressive women had higher concentrations of all SCFAs, except for C6:0. The concentration of isocaproic acid was significantly elevated in subjects with depressive symptoms (median {IQR}: 0.89 {1.15} vs. 0.56 {0.95}, *p* < 0.0001). In non-depressed women, the content of acetic acid was found to be about 15% elevated compared to depressed participants (median {IQR}: 34.99 {19.55} vs. 29.49 {20.81}; *p* = 0.04). Also, a statistical tendency toward higher level of propionic acid (median {IQR}; 21.64 {12.17} vs. 16.88 {9.73}, *p* = 0.07) and C6:0 (median {IQR}: 3.23 {2.33} vs. 1.94 {3.42}, *p* = 0.09) was noticed. The results were replicated regarding the percentage of SCFAs with additional significant differences received for linear C6:0. Results are placed in Table 2.

To evaluate whether the SCFAs content is related to the severity of depressive symptoms, we performed a correlation analysis using a total score of BDI-I and concentration of SCFAs. We found negative correlations between acetate and propionate and Beck’s score (r = −0.21, *p* = 0.02; r = −0.21, *p* = 0.02). A statistical tendency toward a negative correlation between linear valeric acid and BDI-I score (r = −0.17; *p* = 0.07) was noticed. Also, as BDI covers both cognitive/affective and somatic scores we tested whether somatic symptoms might have been overreported in our study group. Consequently, scores on BDI items 1–14 (mood, pessimism, sense of failure etc.) and 15–21 (work inhibition, sleep disturbance, weight loss, loss of appetite etc.) were summed to calculate cognitive/affective and somatic symptom scores, respectively. We found statistically significant correlations between acetate and propionate and BDI somatic score (r = −0.21, *p* = 0.01; r = −0.17, *p* = 0.03), as well as correlations regarding isocaproic and both cognitive/affective (r = 0.37, p = 0.0001) and somatic (r = 9.37, *p* < 0.001) scores. Results are shown in Table 3.

### 3.3. SCFAs Concentration is Modulated by Pharmacotherapy

We did not find statistically significant differences regarding current and past intake of particular medications in terms of depression, however we discovered that pharmacotherapy is a relevant confounder toward fatty acid production in the intestine. Due to small number of participants who declared taking drugs, the statistical power of these associations was below the recommended level of 0.8, so these results must be cautiously analyzed. We noticed that the intake of thyroid drugs in the past affected the level of acetic acid (Users; median {IQR}: 23.07 {12.80} vs. non users 33.73 {21.44}; *p* = 0.041), and current usage of lipid-lowering drugs was associated with the concentration of C6:0 i (Users; median {IQR}: 1.91 {3.62} vs. non-users; 0.55 {0.67}; *p* = 0.0048). No other statistically significant results were obtained.

We also performed a multivariable regression analysis with SCFAs as dependent variables and depression status and the use of drugs as the response variables. Depression and the use of thyroid drugs were independently associated with the level of acetic acid (b = −0.18, *p* = 0.033; b = −0.39, *p* = 0.043, respectively). Depression and the use of lipid lowering drugs were independently associated with the concentration of C6:0i (b = 0.70, *p* = 0.00004; b = 1.02, *p* = 0.0002, respectively). Results are shown in Table 4.

### 3.4. Fiber Intake does not Correlate with SCFAs Concentration and BDI Score

The mean consumption of fiber was 19.01 ± 11.09 g/day (median: 17.39; Min: 1.85; Max: 60.89 g/day). We found that the concentration of SCFAs was not significantly correlated with fiber consumption (Table 5).

We found that women with depression ingested less fiber in comparison to no-depressive participants, however the differences were not statistically significant (Median {IQR}: 13.92 {13.31} vs. 18.73 {10.12}, *p* = 0.1). Similarly, we did not find a correlation between fiber intake and BDI score (r = −0.13; *p* = 0.12).

## 4. Discussion

The microbiota–gut–brain axis has been recently found to mediate the genesis of neuropsychiatric diseases [15,29,30,31,32,33]. A few studies demonstrated depressive-like bacterial fingerprints, however with some contradictions [12,13,14,20,34,35,36,37]. In our study we indirectly looked for the link between microbiota byproducts and depression. Such a strategy is a relatively novel approach, as we found very few studies regarding that issue. To the best of our knowledge, this is the first Polish study investigating the association between gut microbiota SCFAs synthesis and depression in women around the age of 50 years. The present study found that SCFAs, predominantly acetic, propionic, and caproic acids, at least partly contribute to the origin of depressive symptoms. We assessed that medications but not fiber intake are main confounders of SCFAs in our study group.

In our study, we found that median content of acetate was significantly lowered in depressive women compared to non-depressive ones (median: 29.42 µmol/g vs. 34.99 µmol/g; *p* = 0.04). Depressed women also tended to produce less propionic acid (16.88 µmol/g vs. 21.64 µmol/g; *p* = 0.07). In contrast, isocaproic acid concentration was significantly elevated in comparison to subjects with no depressive symptoms (0.89 µmol/g vs. 0.56 µmol/g, *p* < 0.0001). When we conducted Spearman correlation analyses, we found that the concentration of acetic, propionic, and isocaproic acids were negatively correlated with BDI score.

Our study results differ from those obtained by Szczesniak et al. [23] who reported that increased levels of propionic, isobutyric, and isovaleric acids were linked to the depressive patients, the latter one directly and significantly correlated with depression diagnosis, as well as OTU previously linked to the disease [24]. In contrast, Kelly et al. [13] found no differences in major SCFAs between patients diagnosed with MDD and matched controls. More recently, Michels et al. [34] demonstrated that emotional problems in Belgian children were associated with significantly higher concentration of butyrate, isobutyrate, valerate, and isovalerate. Apart from methodological differences, i.e., preparing samples for gas chromatography, different depression diagnostic approaches, and various descent, the discrepancies may be associated with various dietary patterns of study participants, as discussed later. Also, in our study, we evaluated SCFAs content in women only, and not in males or children and the studies on the impact of age and gender on microbiota composition and SCFAs synthesis do exist [35,36].

Acetate was found to be responsible for the prevention of enteropathogenic infection [37], thus maintaining gut barrier integrity, critical for the proper signaling within gut–brain axis [38,39]. Importantly, acetic acid may be utilized by butyrate-producing bacteria to synthesize butyric acid [40,41]. Therefore, lowered content of acetic acid in depressed women form our study group could have influenced the level of butyric acid, found to inhibit the histone deacetylation processes which is of particular interest as previously epigenetic regulation [42] was demonstrated to play a role in hippocampal microglial activation and consequently in depressive-like behaviors secondary to neuro-inflammation [43]. Of note, all major SCFAs are able to act as histone deacetylases inhibitors, although butyrate’s mode of action is the most relevant.

Propionate was recently demonstrated to soften innate immune cells response toward bacterial stimulation, by means of depleted cytokine and nitric oxide production, and did not influence both passive and natural immunization [44]. Therefore, its higher concentrations compared to butyrate may be due to its relatively weak anti-inflammatory activity. Importantly, there is evidence that propionate stimulates the growth of *Bifidobacterium* genus [45], thus contributing to proper permeability of intestinal barrier. A tendency of lower propionic acid level and a negative correlation with BDI score may therefore be the consequence of dysbiosis, thus inflammation in situ as well as within the CNS. Of note, Nankova et al. [46] reported that propionate has the potential to skew neurotransmitter signaling, inflammation, and oxidative stress, all found to be involved in depression pathophysiology [47,48].

As far as caproic acid is concerned, we found little evidence of its biological role [18]. Despite relatively low production of hexanoate, Norin et al. [49] detected the molecule in a quarter of 1-month-old healthy infants. Earlier, it was found that the presence of C6:0 acid is a biomarker of *Clostridium difficile* presence within the gut microbiota [50], which is of a physiological nature in infant gut [51]. However, iso-caproic acid was also detected in child allergy [52], the entity previously reported to be a result of impaired gut microbiota composition [53].

As we used BDI score, which covers not only core depression symptoms but also, among others, sleep disturbance, fatigability, and loss of appetite or weight, all of which may have somatic origin, we correlated BDI cognitive/affective domain and somatic domain with SCFAs concentration. We found statistically significant correlations between C2:0 and C3:0 and BDI somatic score (r = −0.21, *p* = 0.01; r = −0.17, *p* = 0.03), as well as correlations regarding isocaproic and both cognitive/affective (r = 0.37, *p* = 0.0001) and somatic (r = 9.37, *p* < 0.001) scores. Such somatic overlap has already been reported in depression [54,55] and other clinical entities [56,57]. In the case of SCFAs content, it seems that both acetate and propionate are influenced by somatic factors as these two represent the majority of SCFAs, and thus are the first ones to be modulated by energy intake and fiber intake (BDI items: loss of appetite, weight gain) [17]. Unfortunately, we did not collect anthropometric data to expand these associations. Collectively, following other authors [54,55], we confirm that somatic symptoms may essentially influence depressive mood.

Diet is one of the strongest factors influencing gut microbiota composition [58]. It was elegantly evaluated that high-quality diet, regardless of its type, may significantly lower the risk of depression genesis [59]. On the other hand, depression has been found to be associated with nutrition [60]. As reported by Jacka, the consumption of vegetables, fruit, meat, fish, and whole grains was found to diminish the risk for both depression and anxiety disorder [61], via affecting genetic, biochemical, and neurodegenerative factors playing roles in depression origin [62]. Also, the results of currently published study acknowledged the importance of nutritional consulting as an integrative part of mental disease treatment [63], but as emphasized recently better quality nutrition concerned patients with a history of depression, not newly diagnosed [64]. Importantly, SCFAs are synthesized from dietary fiber [65], therefore, we estimated the fiber intake in our study group and found that mean ingestion of fiber was 19.01 ± 11.09 g/day. Although we did not used dietary recall diaries to evaluate the real intake of fiber, our estimates are different from worldwide recommendations [66,67]. Consequently, we did not observe any association between fiber intake and depression, as well as correlation regarding BDI score. The evidence is that a diet with number of fiber-rich products elevates the synthesis of SCFAs [68,69], although the most recent study reported the opposite, however using in vitro model [70]. One study found that during high fiber diet, the populations of *Bacteroidaceae* and *Bifidobacteriaceae* increased and elevated the level of acetate and propionate levels but decreased butyrate content in cecum and blood [71]. Contradictory, high fiber treatment in polyposis mice elevated the counts of both acetate and butyrate-producing bacteria, namely *Bifidobacterium* and clostridial cluster XIVa, *Lachnospiraceae*, and *Anaerostipes*. We did not conduct analyses in a control group with proper intake of fiber, which meant we could not draw conclusions in this respect.

We found that women who declared current usage of lipid-lowering drugs and thyroid medications in the past had lower content of C2:0 and higher of C6:0 i, respectively. Recently, Maier et al. [72] analyzed the potential of over 1.000 commonly used non-antibiotic drugs toward their antimicrobial activity thus implicated into the gut ecosystem dysbiosis. The authors found that 2 out of 3 thyroid agents they analyzed inhibited the growth of particular bacteria strains. Statins have been demonstrated to induce type 2 diabetes mellitus in mice via gut microbiota alterations [73]. Also, Liu et al. [74] demonstrated that treatment responders had increased alpha diversity, with elevated abundances of butyrate producers, namely *Ruminococcaceae*, *Lachnospiraceae*, *Clostridiaceae-1*, and lowered numbers of *Bacteroidetes*. Also, elevated numbers of *Lactobacillaceae* and *Bifidobacteriaceae* in these subjects were found. However, we did not obtain information on the efficacy of treatment in our study group. The results we obtained are therefore possibly due to microbiota alterations, however further studies on the effect of these classes of drugs will elucidate the impact of lipid-lowering and thyroid agents on SCFAs production.

Our study has a few limitations. Our hypothesis on the altered SCFAs within the course of depression is partially based on in vivo experiments. However, studies in rodents widely utilizing behavioral tests, e.g., forced swim test, to induce depression-like behavior [75] might not be appropriate for evaluating such phenotypes. A few authorities have elegantly demonstrated that these “depressive” animal models represent a physiological (adaptive and energy conserving) behavioral style and thus may be encouraged for the research on the mechanism of coping and adaptation to acute stressor, not necessarily for depression [76,77,78,79]. Also, we did not conduct microbiota analysis and its metabolic functions. However, a study evaluating gut barrier integrity is currently running at our research center. The study group consisted of relatively small number of participants. Also, as we detected small number of subjects with moderately heavy and severe depression, we decided to pool depressive participants into one group to elevate the statistical power of our study. Such operation, however, could have influenced the study results. We assume that a more homogenous group of depressive persons, with more pronounced clinical phenotype, would provide more evident results. We evaluated women’s emotional state by means of only BDI score. Nonspecialized questionnaires were used by psychiatric authorities, which possibly could result in false negative results.

## 5. Conclusions

In conclusion, we report that SCFAs may at least partly contribute to women’s emotional health, whereas pharmacotherapy of hyperlipidemia and thyroid disease can affect their synthesis. However, not all available results are consistent with the hypothesis we assumed and as have been suggested by others. As very few studies evaluated the content of SCFAs in neuropsychiatric disorders, among them depression, there is an urgent need to conduct more prospective studies on the involvement of microbiota metabolites on gut–brain axis signaling.

## Figures and Tables

**Table 1:** Sociodemographic and health-related characteristics of study group.

Variable	Depressed *	Non-Depressed *	*P*
Education (primary/vocational/secondary/high)	1/2/13/31	1/7/21/34	0.47
Place of residence (village/town up to 10.000 inhabitants/city up to 100.000 inhabitants/city > 100.000 inhabitants)	3/1/7/34	9/0/2/52	0.51
Marital status (single/married)	12/35	14/49	0.85
Professional activity (yes/no)	42/5	59/4	0.69
Stimulants (alcohol/coffee/coffee+alcohol/smoking/smoking+coffee)	0/21/8/2/8	1/24/8/2/9	0.91
Current Antipsychotic drugs (yes/no)	1/46	1/68	0.99
Current lipid-lowering drugs (yes/no)	4/43	8/61	0.59
Current thyroid drugs (yes/no)	12/35	8/61	0.09
Current hypertension drugs (yes/no)	12/35	14/55	0.51
Antipsychotic drugs in the past (yes/no)	2/45	5/64	0.79
Hypertension drugs in the past (yes/no)	6/41	12/57	0.49
Lipid-lowering drugs in the past (yes/no)	2/45	0/69	0.08
Thyroid drugs in the past (yes/no)	3/44	3/66	0.63
Cardiovascular drugs in the past (yes/no)	6/41	10/59	0.79
Biochemical parameters	Mean ± SD		*p*
Total cholesterol (mg/dL)	208.73 ± 34.24	206.08 ± 29.32	0.66
LDL (mg/dL)	119.99 ± 29.60	116.22 ± 27.71	0.49
HDL (mg/dL)	67.09 ± 18.32	69.77 ± 18.89	0.45
TAG (mg/dL)	108.33 ± 49.10	100.26 ± 53.50	0.42
Fasting glucose (mg/dL)	88.37 ± 14.05	88.09 ± 29.64	0.95
Insulin (µIU/mL)	9.81 ± 3.93	9.31 ± 5.12	0.58
CRP (mg/L)	3.87 ± 9.51	1.85 ± 1.37	0.09

* the total number of subjects depend on data provided within the survey.

**Table 2:** SCFAs content in the depressed and non-depressed group.

SCFA (µmol/g)	Depressed (*n* = 47)		Non-Depressed (*n* = 61)		*P*	R
	Median	IQR	Median	IQR		
C2:0	29.49	20.81	34.99	19.55	0.04	0.19
C3:0	16.88	9.73	21.64	12.17	0.07	0.17
C4:0 i	4.66	2.17	4.94	2.76	0.70	0.04
C4:0 n	20.77	10.52	21.50	19.99	0.68	0.04
C5:0 i	6.99	4.29	7.34	4.53	0.40	0.08
C5:0 n	5.72	2.72	5.83	5.08	0.56	0.05
C6:0 i	0.89	1.15	0.56	0.95	<0.0001	−0.39
C6:0 n	3.23	2.33	1.94	3.42	0.09	−0.16
Total	94.04	47.05	103.79	72.41	0.17	0.12
SCFAs (%)	Median	IQR	Median	IQR	*p*	
C2:0	31.04	11.25	33.91	9.10	0.05	0.18
C3:0	18.56	3.94	19.77	5.58	0.14	0.13
C4:0 i	5.66	2.06	5.01	2.70	0.34	−0.09
C4:0 n	21.38	6.95	21.34	7.57	0.29	−0.12
C5:0 i	8.10	4.20	8.16	5.25	0.85	−0.02
C5:0 n	6.22	2.30	6.13	1.83	0.29	−0.10
C6:0 i	0.91	1.38	0.42	0.46	<0.0001	−0.40
C6:0 n	3.22	3.07	2.41	3.08	0.03	−0.20

r—effect size statistic for the Mann–Whitney test.

**Table 3:** Correlation between SCFAs concentration and BDI-I score.

BDI (Total Score)			BDI (Cognitive/Affective Score)		BDI (Somatic Score)	
SCFA	r	*p*	r	*p*	r	*p*
C2:0	−0.20	0.03	−0.09	0.18	−0.21	0.01
C3:0	−0.21	0.02	−0.1	0.15	−0.17	0.03
C4:0 i	−0.07	0.44	0.07	0.6	−0.02	0.57
C4:0 n	−0.10	0.28	0.04	0.85	−0.02	0.58
C5:0 i	−0.10	0.26	0.03	0.91	−0.07	0.28
C5:0 n	−0.17	0.07	−0.006	0.72	0.05	0.41
C6:0 i	0.12	0.19	0.37	0.0001	0.37	<0.001
C6:0 n	−0.04	0.63	0.19	0.07	0.15	0.15
Total	−0.14	0.09	−0.04	0.47	−0.14	0.06

r—correlation coefficient, BDI—Beck’s Depression Inventory.

**Table 4:** Linear regression of SCFAs on depression status, the use of lipid lowering drugs and thyroid drugs.

SCFAs	Depression				Lipid-Lowering Drugs				Thyroid Drugs			
	b	SE	*t*	*p*	b	SE	*t*	*p*	b	SE	*t*	*p*
C2:0	−0.18	0.09	−2.16	0.033	−0.24	0.14	−1.77	0.080	−0.39	0.19	−2.04	0.043
C3:0	−0.16	0.09	−1.78	0.078	−0.16	0.14	−1.14	0.258	−0.43	0.19	−2.20	0.030
C4:0 i	−0.03	0.08	−0.40	0.690	0.12	0.12	0.97	0.333	−0.01	0.17	−0.07	0.941
C4:0 n	0.0007	0.12	0.006	0.996	−0.06	0.19	−0.31	0.756	−0.32	0.27	−1.20	0.233
C5:0 i	−0.07	0.09	−0.79	0.432	0.10	0.15	0.65	0.516	0.05	0.21	0.23	0.819
C5:0 n	−0.05	0.09	−0.51	0.608	0.13	0.14	0.93	0.355	−0.14	0.19	−0.71	0.480
C6:0 i	0.70	0.16	4.28	0.00004	1.02	0.26	3.87	0.0002	0.37	0.36	1.02	0.310
C6:0 n	0.27	0.16	1.71	0.090	0.38	0.25	1.50	0.135	0.13	0.35	0.39	0.700
Total	−0.10	0.07	−1.34	0.184	−0.06	0.12	−0.48	0.632	−0.29	0.16	−1.76	0.082

b—regression coefficient, SE—standard error of the regression coefficient, t—t-statistic, SCFAs—short chain fatty acids.

**Table 5:** The correlation between fiber intake and SCFAs level.

Fiber Intake vs.	R	*P*	95% Confidence Interval
C2:0	0.07	0.45	−0.15; 0.21
C3:0	0.038	0.69	−0.19; 0.17
C4:0 i	0.065	0.49	−0.16; 0.20
C4:0 n	0.0004	0.99	−0.23; 0.13
C5:0 i	0.10	0.27	−0.16; 0.21
C5:0 n	−0.007	0.93	−0.21; 0.15
C6:0 i	−0.08	0.38	−0.25; 0.11
C6:0 n	−0.02	0.85	−0.21; 0.16
Total	0.03	0.72	−0.19; 0.17
